# Reduced Humoral Response of SARS-CoV-2 Antibodies following Vaccination in Patients with Inflammatory Rheumatic Diseases—An Interim Report from a Danish Prospective Cohort Study

**DOI:** 10.3390/vaccines10010035

**Published:** 2021-12-28

**Authors:** Karen Schreiber, Christine Graversgaard, Randi Petersen, Henning Jakobsen, Anders Bo Bojesen, Niels Steen Krogh, Bente Glintborg, Merete Lund Hetland, Oliver Hendricks

**Affiliations:** 1 Danish Hospital for Rheumatic Diseases, 6400 Sønderborg, Denmark; kschreiber@danskgigthospital.dk (K.S.); CGraversgaard@danskgigthospital.dk (C.G.); RPetersen@danskgigthospital.dk (R.P.); HJakobsen@danskgigthospital.dk (H.J.); anders@socioskop.dk (A.B.B.); nielssteenkrogh@zitelab.dk (N.S.K.); 2 Department of Regional Health Research (IRS), University of Southern Denmark, 5230 Odense, Denmark; 3 Thrombosis and Haemostasis, Guys and St Thomas’ NHS Foundation Trust, London SE1 7EH, UK; 4 DANBIO, The Danish Rheumatologic Biobank and Copenhagen Center for Arthritis Research (COPECARE), Center for Rheumatology and Spine Diseases, Centre of Head and Orthopedics, Copenhagen University Hospital Rigshospitalet, 2100 Glostrup, Denmark; glintborg@dadlnet.dk (B.G.); merete.hetland.01@regionh.dk (M.L.H.); 5 Department of Clinical Medicine, Faculty of Health and Medical Sciences, University of Copenhagen, 1165 Copenhagen, Denmark

**Keywords:** COVID-19 vaccine, RMD, humoral response

## Abstract

**Background/Purpose:** In light of the current COVID-19 pandemic, whether patients with rheumatic musculoskeletal disease (RMD) treated with conventional (cs) or biologic (b) disease-modifying drugs (DMARDs) exhibit an adequate immune response to the currently available SARS-CoV-2 vaccinations remains a major concern. There is an urgent need for more SARS-CoV-2 vaccine efficacy data to inform healthcare providers on the potential need for a booster vaccine. We established the ‘*Detection of SARS-CoV-2 antibodies in Danish Inflammatory Rheumatic Outpatients*’ study (DECODIR) in March 2021 in order to assess and compare the immunoglobulin G (IgG response) of the SARS-CoV-2 BNT162b2 vaccine (Pfizer, Groton, CT, USA/BioNTech, Mainz, Germany) and mRNA-1273 vaccine (Moderna, Cambridge, MA, USA) administered as part of the national vaccine roll out in patients with RMDs, irrespective of treatment. Patients’ SARS-CoV-2 IgG level was used as proxy to determine vaccination response. **Methods:** The study is a longitudinal prospective cohort study in which the SARS-CoV-2 antibody response was measured and compared at baseline and at six weeks following vaccination. The study population consisted of patients with rheumatoid arthritis (RA), spondyloarthropathies (SpA), or psoriatic arthritis (PsA) receiving their outpatient treatment at the Danish Hospital for Rheumatic Diseases, Sonderborg. Bloods, patient reported outcome measurements (PROMS), clinical data, and treatment information (cs/bDMARD) were collected at baseline/6 weeks and documented in the Danish DANBIO registry. Commercially available antibody tests (ThermoFisher, Waltham, MA, USA) were used, and SARS-CoV-2 IgG levels were reported in EliA U/mL. Sufficient IgG response was defined as ≥10 EliA U/mL (manufacturers cut-off). Associations between antibody response, age, gender, disease (RA/PsA/SpA), no treatment or cs/bDMARD treatment, and disease activity were tested using proportional odds regression and bootstrapped tests of medians. Results were reported using mean, median (IqR), and bootstrapped 95% confidence interval (CI) of the median. **Results:** A total of 243 patients were included. We observed a significant increase in IgG levels (median of <0.7 EliA U/mL at baseline versus 34.5 EliA U/mL at 6 weeks). Seventy-two patients (32%) had an insufficient IgG response. The median IgG level in patients treated with cs/bDMARD combination therapy was significantly lower compared to patients without any DMARD treatment (12 EliA U/mL vs. 92 EilA U/mL (*p* < 0.01)). **Conclusion:** Patients treated with a combination of cs/bDMARD are at significantly higher risk of an inadequate response to SARS-CoV-2 vaccines as measured by IgG level compared to patients without DMARD treatment. IgG SARS-CoV-2 are only part of the immune response, and further data are urgently needed. At present, our results may inform healthcare providers and policy makers on the decision for the need of a booster vaccine in this particular patient group.

## 1. Introduction

The COVID-19 pandemic has resulted in almost 4 million deaths globally, and the availability and consistent application of effective SARS-CoV-2 vaccines is the cornerstone to controlling the pandemic.

Rheumatic musculoskeletal diseases (RMD) are mainly chronic, systemic inflammatory conditions, and most patients require long-term treatment with immosuppressive medications by means of either conventional synthetic (cs) and/or biologic (b) disease-modifying antirheumatic drugs (cs/bDMARDs). Some patients with RMDs, in particular those with SpA, may also only receive nonimmune-suppressant medication, such as non-steroidal anti-inflammatory drugs and physiotherapy (subsequently referred to as ‘no DMARD’). 

One apparent concern for rheumatologists and patients with RMDs remains the scarce and conflicting evidence as to whether patients with RMDs are more likely to contract SARS-CoV-2 infection and if they are more likely to experience a more severe COVID-19 disease course compared to the background population. Previous studies in patients with rheumatoid arthritis (RA) and spondyloarthropathies (SpA) has neither confirmed strong associations to the risk of contracting a SARS-CoV-2 infection, nor confirmed a significantly increased risk of adverse COVID-19-related outcomes such as intensive care unit (ICU) admission, hospitalization, or COVID-19-related death; furthermore, previous COVID-19 risk scenarios represented the pre-vaccination era [1,2]. However, data from large cohort studies in Denmark and the United States suggest that the risk for hospitalization compared to the background population is 30% increased in RA [3] and threefold in the case of patients with systemic lupus erythematosus (SLE) [4]. Data from the U.K. biobank cohort showed that patients with RA are at higher risk of COVID-19-related death compared to the background population [5]. Further data are needed to clarify the general risk the COVID-19 pandemic imposes on patients with RMDs.

A second concern remains as to whether vaccine efficacy data from the original phase III studies on the SARS-CoV-2 vaccines, BNT162b2 vaccine (Pfizer and BioNTech), and mRNA-1273 vaccine (Moderna) are applicable to patients with RMDs. Due to the nature of phase III studies, subjects with confirmed or suspected immunosuppressive or immunodeficiency were excluded. The effectiveness of the vaccines can therefore not be applied to a patient population on DMARDs (conventional, biologic or targeted synthetic) as these patients were not included in the above-mentioned studies [6,7]. 

Data on the efficacy of the BNT162b2 vaccine and mRNA-1273 vaccine in patients with RMDs assessing the effect of cs/bDMARD treatment remain scarce, and the currently available evidence stems mainly from limited-sized cohort studies. While healthy individuals with SARS-CoV-2 infection develop antibodies, with IgG remaining detectable for at least four months [8], patients with RMDs apparently have a reduced humoral immune response after their first vaccine dose [9]. There remains an unmet need for SARS-CoV-2 vaccine efficacy data assessing the vaccine efficacy in this patient group, taking into account factors that may influence the individual vaccine response such as underlying disease and treatment. 

Our main aim of the present ‘*Detection of SARS-CoV-2 antibodies in Danish Inflammatory Rheumatic Outpatients*’ study (DECODIR study) was to assess and compare the IgG response of the SARS-CoV-2 vaccines BNT162b2 vaccine (Pfizer/BioNTech) and mRNA-1273 vaccine (Moderna) administered as part of the national vaccination program in patients with ORMDs according to their current no DMARD or cs/bDMARD-treatment. The level of SARS-CoV-2 IgG in individual patients was used as proxy to determine vaccination response at baseline and after 6 weeks and documented in the Danish DANBIO registry. 

## 2. Methods

The DECODIR study was established in March 2021 in an outpatient clinic at the Danish Hospital for Rheumatic Diseases in Sonderborg. In Denmark, patients with inflammatory rheumatic arthritis (IRD) are monitored prospectively in the nationwide DANBIO database [10]. At each outpatient visit, patient-reported outcome measures (e.g., pain) and impact on daily activities are recorded, as well as objective findings of inflammatory activity (swollen joints), current treatment with predisolon, treatment with no DMARD or cs/bDMARD treatment, and biochemical data (including C-reactive protein, full blood count, and liver function tests).

The Pfizer/BioNTech and Moderna vaccines were administered as part of the Danish national vaccine role out and were provided on the basis of availability in the country; they were not chosen by preference. Availability was dependent on international distribution. 

### 2.1. Patient Eligibility and Consent

All adult patients with RA, PsA, and SpA receiving the SARS-CoV-2 vaccine were invited to participate. Exclusion criteria included: not being able to provide written informed consent, pregnancy wish or ongoing breastfeeding, any contraindication to receive COVID-19 vaccination, and having already been vaccinated for SARS-CoV-2. A total of 1664 patients received the invitation to participate in the study. Two hundred and forty-seven patients were accepted to participate. Four patients had to be excluded, of these four patients two patients were excluded due to personal reasons, one patient failed screening, and one patient was acute hospitalized.

Ethics approval was obtained from the local ethics committee in South Denmark (approval no. 21/11693).

Patients were informed about the study at their usual routine clinical appointment and were invited to take part. Each patient was informed about the study and study method and was given as much time as needed to decide upon participation. 

### 2.2. User Involvement

The project was presented to the research department’s user council in February 2021. The project managers received clear positive feedback from patient representatives. The purpose and organization of the project were found to be highly relevant from the patient perspective. Two project-responsible patient researcher partners were appointed in cooperation with the user council and participated during the course of the project. 

### 2.3. Source Data

Data and outcomes were registered in an electronic Case Report Form (eCRF), based on the Reuma-eCRF system available within DANBIO at each patient visit. 

### 2.4. Antibody Testing

Commercially available antibody tests (ThermoFisher) were used, and SARS-CoV-2 IgG levels were reported in EliA U/mL in 1:100 diluted serum samples. A sample with antibody level ≥10 EliAU/mL is seropositive (according to the manufacturers cut-off). 

### 2.5. Statistics

Associations between antibody response, age, gender, disease (RA/PsA/SpA), no DMARD or cs/bDMARD treatment, vaccine type (Pfizer vs. Moderna), and DAS28-CRP were tested using proportional odds regression and bootstrapped tests of medians. Results are reported using mean, median (IqR), and bootstrapped 95% confidence interval (CI) of the median.

## 3. Results

A total of 243 patients were included, and all patients performed blood samples at baseline and after 6 weeks. Demographics are outlined in Table 1.

We observed a significant increase in IgG levels comparing baseline versus 6 weeks (median of <0.7 EliA U/mL at baseline versus 34.5 EliA U/mL at 6 weeks). 

**Figure 1:**
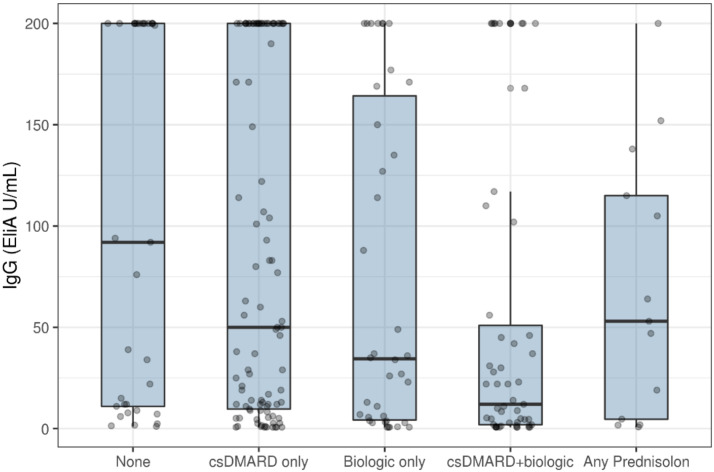
Vaccination response (IgG-level) 6 weeks after initial vaccination stratified by antirheumatic treatment. IgG—immunoglobulin G; DMARD—disease-modifying drugs; csDMARD only—conventional DMARDBox plot showing median and interquartile range. In the interest of space in the figure we referred to ‘biologic DMARD’ as ‘Biologic only’.

Seventy-two patients (32%) had an insufficient IgG response (≤10 EliA U/mL). Median IgG levels in patients on cs/bDMARD combination therapy was significantly lower compared to patients without any DMARD treatment (12 EliA U/mL vs. 92 EilA U/mL (*p* < 0.01)) (Figure 1).

A significant lower median IgG level was found in patients who received Pfizer ((median 32 EliA U/mL (95%CI: 18–50)) compared to Moderna ((69.5 EliA U/mL (95%CI: 28–135)). However, the proportional odds model showed no significant difference (*p* = 0.164).

**Figure 2:**
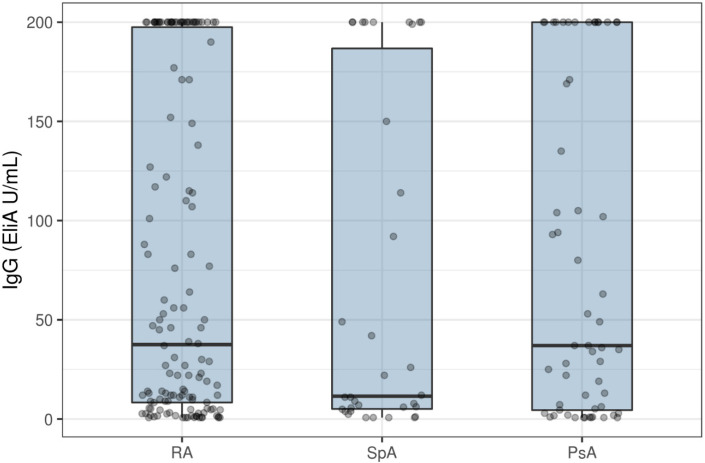
Vaccination response (IgG-level) 6 weeks after initial vaccination stratified by diagnosis. IgG—immunoglobulin G; RA—rheumatoid arthritis; SpA—spondyloarthropathies, PsA—psoriatic arthritis. Box plot showing median and interquartile range.

A significantly lower median IgG level was observed for the SpA group (median 11.5 EliA U/mL (95%CI: 6.3–92)) compared to both the RA (median 37.5 EliA U/mL (95%CI: 19–60)) and PsA groups (median 37 EliA U/mL (95%CI: 22–94)). The proportional odds model showed no significant differences across diagnosis groups (*p* = 0.605) (Figure 2).

## 4. Discussion 

We developed the study outlined above in order to assess the impact of cDMARD and bDMARD on the SARS-CoV-2 humoral vaccine response.

Our data provide further evidence on the reduced humoral response to the SARS-CoV-2 vaccine in patients with RMDs. We found lower levels of IgG in all groups of patients treated with any type of DMARD or prednisolone at any dose, when compared to patients without active treatment. In particularthe combination of csDMARD and bDMARD revealed a significantly higher risk of an inadequate SARS-CoV2 vaccine response. To the best of our knowledge, our study shows for the first time that patients with combination therapy may be less likely to develop an immune response compared to those who are in monotherapy with conventional DMARDs or not receiving active treatment. 

The results contrast the evidence that the applied vector-based and mRNA SARS-CoV-2 vaccines result in robust humoral and cellular immune responses in healthy individuals [8,11,12]. 

Previous observational studies of limited size suggest adequate vaccine response [13], whilst others suggest a potentially reduced immune response [14]. A reduced humoral response after the initial vaccine was evident in a cohort of 120 patients with immune-mediated inflammatory diseases, of which 15% received DMARD therapy. In this cohort, patients who were treated with methotrexate (classified as a conventional DMARD) did not develop detectable SARS-CoV-2 antibodies [9].

Our study has some shortcomings. Firstly, T-cell responses were not measured [15]. It is likely that patients who have been vaccinated with SARS-CoV-2 develop some T-cell response without exhibiting adequate IgG levels. After all, IgG SARS-CoV-2 are only part of the immune response, and further data are needed to assess the specific effect of DMARD combinations on T-cell responses. DMARDs are non-targeted therapies, and their effect on the immune system is pleiotropic [16]. Moreover, our patients were invited to participate, and despite inviting all patients attending in our outpatient service, there may have been some selection bias in that some patient groups may have been underrepresented. This could be patients of a particular age group (i.e., very old patients who struggle to attend clinics), or patients with limited resources or other comorbidities, which may have impacted the possibility of patients attending the serial blood tests. With regards to the former, the age of our patient group is well balanced in those above the age of 50, whilst only 10% of patients were in the age group 30–49. This of course limits the generalizability of our findings.

However, data that further provide evidence on the immune responses to SARS-CoV-2 in potentially vulnerable patient groups are urgently needed. On 4 October 2021, the European Medicines Agency (EMA) aligned their recommendation with the recommendation of the Center for Disease Control and Prevention (CDC) of a third (booster) SARS-CoV-2 vaccine for patients with a ‘*weakened immune system*’ in view that an extra dose would increase the protection level [17,18]. Guidelines across Europe are less stringent, and at present, our results may inform healthcare providers and policymakers on the decision for the need of a booster vaccine in these patients. Further data are needed to shed light on how long IgG antibody levels persist, whether booster vaccines can impact the long-term immune response, and moreover, whether or to which extent our findings are supported by similar T-cell responses. Corresponding analyses of IgG levels and T-cell responses are planned to be performed 26 and 52 weeks after the initial first vaccination.

## Figures and Tables

**Table 1:** Patient baseline characteristics.

Number of Patients, N	243
Men/women	135 (56%)/108 (44%)
Age, years, N (%)	
30–49	24 (10%)
50–59	54 (22%)
60–69	75 (31%)
70+	90 (37%)
Antirheumatic treatment, N (%)	
None	33 (14%)
Conventional synthetic DMARD only	105 (43%)
Biologic DMARD only	41 (17%)
Conventional synthetic DMARD + biologic DMARD	51 (21%)
Prednisolone monotherapy (varying doses)	13 (5.3%)
Diagnosis: RA, n (%)	142 (58%)
SpA, n (%)	39 (16%)
PsA, n (%)	60 (25%)
Vaccine given, n (%)	
Moderna *	25 (10%)
Pfizer	218 (90%)
DAS28crp ^+^, n (%)	
<3	171
3+	27
N/A	45

DMARD—disease-modifying drug; PsA—psoriatic arthritis; RA—rheumatoid arthritis; SpA—spondyloarthritis. Baseline measurements carried out on average at 0.6 days after the first dose was given (SD = 3.7). ^+^ DAS28crp was measured at the last regular visit before the baseline visits. * availability of the vaccine was limited in Denmark.

## Data Availability

The datasets generated during and/or analysed during the current study are available from the corresponding author on reasonable request.
